# *TET2* and *DNMT3A* mutations and exceptional response to 4′-thio-2′-deoxycytidine in human solid tumor models

**DOI:** 10.1186/s13045-021-01091-5

**Published:** 2021-05-26

**Authors:** Sherry X. Yang, Melinda Hollingshead, Larry Rubinstein, Dat Nguyen, Angelo B. A. Larenjeira, Robert J. Kinders, Michael Difilippantonio, James H. Doroshow

**Affiliations:** 1 Division of Cancer Treatment and Diagnosis, National Cancer Institute, National Institutes of Health, Bethesda, MD USA; 2 Leidos Biomedical Research, Inc., Frederick, MD USA

**Keywords:** DNMT3A and TET2 mutations, NCI-H23 cells, p21, 4′-thio-2′-deoxycytidine (T-dCyd), Whole exome sequencing, Xenograft tumors

## Abstract

**Background:**

Challenges remain on the selection of patients who potentially respond to a class of drugs that target epigenetics for cancer treatment. This study aims to investigate *TET2/DNMT3A* mutations and antitumor activity of a novel epigenetic agent in multiple human cancer cell lines and animal models.

**Methods:**

Seventeen cancer cell lines and multiple xenograft models bearing representative human solid tumors were subjected to 4′-thio-2′-deoxycytidine (T-dCyd) or control treatment. Gene mutations in cell lines were examined by whole exome and/or Sanger sequencing. Specific gene expression was measured in cells and xenograft tumor samples by Western blotting and immunohistochemistry. *TET2/DNMT3A* mutation status in 47,571 human tumor samples was analyzed at cBioPortal for Cancer Genomics.

**Results:**

Cell survival was significantly inhibited by T-dCyd in breast BT549, lung NCI-H23, melanoma SKMEL5 and renal ACHN cancer lines harboring deleterious *TET2* and nonsynonymous *DNMT3A* mutations compared to 13 lines without such mutation pattern (*P* = 0.007). The treatment upregulated p21 and induced cell cycle arrest in NCI-H23 cells, and dramatically inhibited their xenograft tumor growth versus wildtype models. T-dCyd administrations led to a significant p21 increase and near eradication of tumor cells in the double-mutant xenografts by histological evaluation. *TET2/DNMT3A* was co-mutated in human lung, breast, skin and kidney cancers and frequently in angioimmunoblastic and peripheral T cell lymphomas and several types of leukemia.

**Conclusions:**

Cell and animal models with concurrent mutations in *TET2* and *DNMT3A* were sensitive to T-dCyd treatment. The mutations were detectable in human solid tumors and frequently occur in some hematological malignancies.

**Supplementary Information:**

The online version contains supplementary material available at 10.1186/s13045-021-01091-5.

## Introduction

The family of DNA methyltransferases (DNMTs) including DNMT3A (NM_022552.4) catalyzes the addition of a methyl group to 5-cytosine residue of CpG dinucleotides. DNMT3A, encoded by *DNMT3A* gene (2p23.3), methylate previously unmethylated regions of genomic DNA and is responsible for genome-wide de novo DNA methylation [1]. DNMT3A also functions as a transcriptional co-repressor that does not require its de novo methyltransferase activity to silence gene transcription [2, 3]. It is frequently mutated in AML and was associated with poor prognosis; however, DNMT3A alteration has not been much described in human solid tumors [4]. Currently, data are mixed regarding the relationship between *DNMT3A* mutations and treatment response to DNMT inhibitors such as decitabine or azacitidine in myeloid malignancies [5–7]. It remains unclear whether *DNMT3A* mutation is associated with antitumor activity of DNMT inhibitors and other epigenetic modulators in human solid tumors and their corresponding animal models.

TET2 (NM_001127208.2) coded by this gene (4q24) is a member of the TET family proteins (TET1–3) that convert 5-methylcytosine to 5-hydroxymethylcytosine in DNA, and promotes site-specific DNA demethylation [8]. In addition to its role in demethylation, TET2 appears to act as a tumor suppressor that is involved in the control of balancing survival, growth and differentiation in normal hematopoiesis. Loss of TET2 induced leukemogenesis in hematopoietic cells [9]. *TET2* mutations, as an oncogenic process, are frequently observed in AML, MDS and lymphoid malignancies [10–12] as well as mutated in some human solid tumors [13]. It has been shown that *TET2* mutations were associated with higher response rate to decitabine and azacitidine therapy in MDS [14]. However, no preclinical and clinical data are available on the association between the *TET2* mutations and antitumor activity of DNMT inhibitors and other epigenetic modulating agents in human solid tumors. In addition, *DNMT3A* and *TET2* mutations occur concurrently in human malignancies such as T cell lymphoma [15, 16]. Double-gene knockout led to the transformation of hematopoietic stem cells through blocking cellular differentiation [17].

A novel epigenetic modulator 4′-thio-2′-deoxycytidine (T-dCyd) demonstrated antitumor activity in vivo although it was unclear for its mechanisms of action [18], and is currently in early clinical development. In this study, we hypothesized that aberration of the genes within the epigenetic regulatory network is critical to T-dCyd antitumor activity. The hypothesis was examined through T-dCyd treatment of multiple cancer cell lines and xenograft models with and without relevant gene mutations implicated in the epigenetics. We also investigated *TET2/DNMT3A* mutation frequencies in over 100 cancer types using cBioPortal for Cancer Genomics (www.cbioportal.org) [19].

## Methods

### Drugs, study model cell lines and drug treatment

Lung adenocarcinoma NCI-H23 and EKVX, colorectal adenocarcinoma COLO205, HCT15, HCT116 and KM12, ovarian carcinoma SKOV3 and OVCAR3, and melanoma SKMEL2, SKMEL5 and M14, breast cancer MCF7, T47D, MDA-MB-231 and BT549, renal cell adenocarcinoma ACHN and prostate cancer DU145 cell lines were obtained from the Tumor/Cell Line Repository, Division of Cancer Treatment and Diagnosis, National Cancer Institute (Frederick, MD). T-dCyd was obtained from the Developmental Therapeutics Program, Division of Cancer Treatment and Diagnosis, National Cancer Institute (Rockville, MD). They were dissolved in 0.05% Tween 80 saline and reconstituted to 10 mM; aliquots of the drug were stored at − 80 °C until use.

### *DNMT3A/TET2* mutations by WES in cancer cells and human malignancies

Genomic DNA extraction in cancer cell lines was performed using Omega Biotek nucleic acid isolation kit per the manufacturer instructions (Omega Bio-tek, Norcross, GA). DNA concentration was measured with use of Promega QuantiFluor dsDNA System on a Quantus Fluorometer (Promega, Madison, WI). The integrity of DNA was analyzed using the Genomic DNA Screen Tape on an Agilent 2200 TapeStation instrument (Agilent Technologies, Santa Clara, CA). The whole exome sequencing (WES) was carried out by Illumina Nextera Rapid Capture Exome sequencing following the standard Illumina kit protocol (Illumina, San Diego, CA). Briefly, 50 ng of genomic DNA was fragmented, and barcoded linkers were ligated to generate indexed libraries. These were hybridized to human exome probe pool to capture and enrich for exome sequences. The exome libraries were quantified using Promega QuantiFluor dsDNA System, and size and purity of the libraries were analyzed by High Sensitivity D1000 Screen Tape on the Agilent 2200 TapeStation instrument. The libraries were pooled and run on an Illumina HiSeq 2500 sequencer utilizing the paired end 100 bp rapid run format to generate an average of 30 × sequencing coverage per sample. The raw FASTQ data were adaptor-trimmed and mapped to hg19 human reference genome with the BWA Enrichment software, and specific variants were annotated using the Variant Studio software within the Illumina BaseSpace applications suite (www.basespace.illumina.com).

*TET2* and *DNMT3A* mutations in a curated set of the non-redundant studies including 47,571 human tumor samples were analyzed at cBioPortal for Cancer Genomics [19]. The data were lastly accessed on December 26, 2020.

### PCR and Sanger sequencing

Primers were designed to amplify ~ 300 bp genomic sequence flanking the mutation of interest of *TET2* gene. The primers that cover the *TET2* c.5162T > G mutation in exon 11 were 5′-AGTCTCAGCCGATGGATCTG-3′ (forward) and 5′-AGGGCATGAAGAGAGCTGTT-3′ (reverse). PCR was carried out using the Kapa HiFi HotStart PCR kit (Kapa Biosystems, Wilmington, MA), and the amplicons were purified by the Mag-Bind® RxnPure Plus reagent (Omega Bio-tek). Each amplicon was sequenced with forward and reverse primers using the Big Dye chemistry on an ABI 3130 capillary genetic analyzer (Applied Biosystems, Foster City, CA).

### Immunohistochemistry or immunocytochemistry and quantitative analysis

Immunohistochemistry on the formalin-fixed and paraffin-embedded sections was described previously [20–22]. In brief, rabbit monoclonal antibodies to DNMT3A (clone D23G1) and CDKN1A/p21^Waf1/Cip1^ (p21, clone 12D1) were obtained from Cell Signaling Technology (Danvers, MA), and rabbit polyclonal antibodies to TET2 (ABE364) was purchased from EMD Millipore (Burlington ​, MA). They were added to slides in dilutions of 1:50 for DNMT3A and p21, and 1:1000 for TET2. The binding of antibodies to their antigenic sites in sections was amplified using Vectastain Elite avidin–biotin-peroxidase complex kits (Vector Laboratories, Burlingame, CA). The antigen–antibody reaction sites were visualized using 3,3-diaminobenzidine for 7 to 10 min and, subsequently, sections were counterstained with Mayer**’**s hematoxylin. Formalin-fixed and paraffin-embedded MCF-7, HCT116 treated with 10 nM of topotecan and NCI-H23 cells were used as positive controls for DNMT3A, p21 and TET2, respectively. Areas of tumor cell staining on each tumor sample were analyzed with the assistance of a digital imaging system (DAKO, Carpinteria, CA) reporting the intensity and percentage of staining for DNMT3A, and TET2 to determine the staining Index (SI). It was calculated as the percentage multiplied by intensity of staining (after subtracting the tissue readout of the corresponding negative control) divided by 100 (SI = intensity × percentage/100) [20]. p21 was reported as the percentage of tumor cell staining as described previously [23].

### Clonogenic assay

After 48 h of drug treatment, 2000 cells were washed and plated into 6-well plates with each condition in triplicate as described previously [24]. After 10–14 days, colonies were fixed in 10% methanol 10% acetic acid glacial solution and, following wash, stained with 0.1% crystal violet in plates. The colonies formed were counted using an automated ACCU count TM 1000 (Biologic Inc., Manassas, VA). The fraction of colony formation (growth) relative to vehicle-treated control or of growth inhibition was calculated by: (average of drug-treated)/vehicle-treated) × 100 or as: [1 − (average of drug-treated)/vehicle-treated)] × 100.

### Western blotting

Cell pellets were lysed in 1 × sample buffer containing phosphatase and protease inhibitors. The lysates were sonicated, quantified and boiled for 10 min. Equal amount was electrophoresed in 7.5% or 4–20% SDS–polyacrylamide gels (Bio-Rad, Hercules, CA, USA). After transferring the proteins on to nitrocellulose membrane, filters were incubated in 5% non-fat dry milk in PBST (1X PBS plus 0.2% Tween-20) for 1 h. Blot was probed with p21 antibody (1:1000 dilution, Cell Signaling Technology, Danvers, MA) or TET2 antibody (EMD Millipore) in 1:2000 dilution and ß-actin antibody (1: 10,000 dilution) overnight at 4 °C in PBST, and subsequently incubated with a horseradish peroxidase-conjugated secondary antibody (BioRad) for 1 h at room temperature. Immuno-bound antibodies were detected by Super Signal West Pico Chemiluminescent Substrate detection reagent (Pierce/Thermo Scientific, Rockford, IL, USA) and visualized by autoradiography. The band density on the scanned images was quantified by ImageJ software (ImageJ.net).

### Cell cycle analysis

Cells were treated with different concentrations of T-dCyd and vehicle. After 24 h of treatment, they were detached with AccutaseTM solution (Millipore), washed twice with PBS and fixed in cold 70% ethanol overnight. Following washing, fixed cells were stained with 1 ml of propidium iodide solution (50 µg/ml) supplemented with 50 µl RNaseA (50 mg/ml) at 37 °C for 1 h and analyzed with a FACSCanto II Flow Cytometer (Becton Dickinson, Franklin Lakes, NJ). After collecting at least 20,000 cells, the cell cycle profile was analyzed using the FlowJo software (Becton Dickinson).

### TUNEL assay

The assay (R&D Systems, Minneapolis, MN) measures the fragmented DNA in cells undergoing apoptosis [20]. DNA breaks at 3′-DNA ends were labeled with biotinylated nucleotides catalyzed by terminal deoxynucleotidyl transferase. An avidin-conjugated horseradish peroxidase (Vector Laboratories Inc.) specifically bound to the biotinylated DNA fragments and produced a brown precipitate in the presence of 3,3-diaminobenzidine. A tumor specimen treated by nuclease was used as a positive control, and the same tumor section without nuclease treatment was used as a negative control.

### Human xenograft tumor establishment and drug administration

Female athymic nude mice (nu/nu NCr; Animal Production Program, National Cancer Institute at Frederick, Frederick, MD, USA) were implanted by subcutaneous injection of a cell suspension of human cancer cells into the flank tissues as described previously [25]. The mice were subjected to the treatment groups for each model when the tumors reached a median tumor staging size less than 200 mg in weight. They were subsequently treated with T-dCyd at 4 mg per kg, PO or with saline in mice bearing NCI-H23 (or IP) and SKOV3 tumors on a weekly schedule with daily dosing for 5 days for 3 cycles, and M14 for 4 cycles and COLO205 for 2 cycles. Tumor size was measuredly weekly during treatment. The National Cancer Institute Frederick National Laboratory for Cancer Research is accredited by the Association for Assessment and Accreditation of Laboratory Animal Care International and observes the US Public Health Service Policy on Care and Use of Laboratory Animals. All the experiments were conducted per an approved animal care and use committee protocol in accordance with the procedures outlined in the Guide for Care and Use of Laboratory Animals, Eighth Edition (National Research Council, 2011; National Academy Press; Washington, D.C.).

### Statistical analysis

Unpaired student *t* test was used to assess the difference in tumor sizes on the H&E slides, and expression of DNMT3A, TET2 and p21 between T-dCyd and saline treatments in NCI-H23 xenograft tumors. The unpaired *t* test was also utilized to evaluate the cell growth inhibition by T-dCyd between cell lines with co-occurrence of *TET2/DNMT3A* mutations and without such pattern of mutations, as well as growth inhibition between vehicle- and T-dCyd-treatments in NCI-H23 and SKOV3 cells. p21 expression between T-dCyd at different concentrations and vehicle treatment in NCI-H23 cells was evaluated using one sample *t* test (GraphPad Prism version 8.4.3). All statistical tests were two-sided, and the significance level was pre-specified with a *P* value of < 0.05.

## Results

### *TET2* and *DNMT3A* mutations in cancer cell lines and human malignancies

**Fig. 1:**
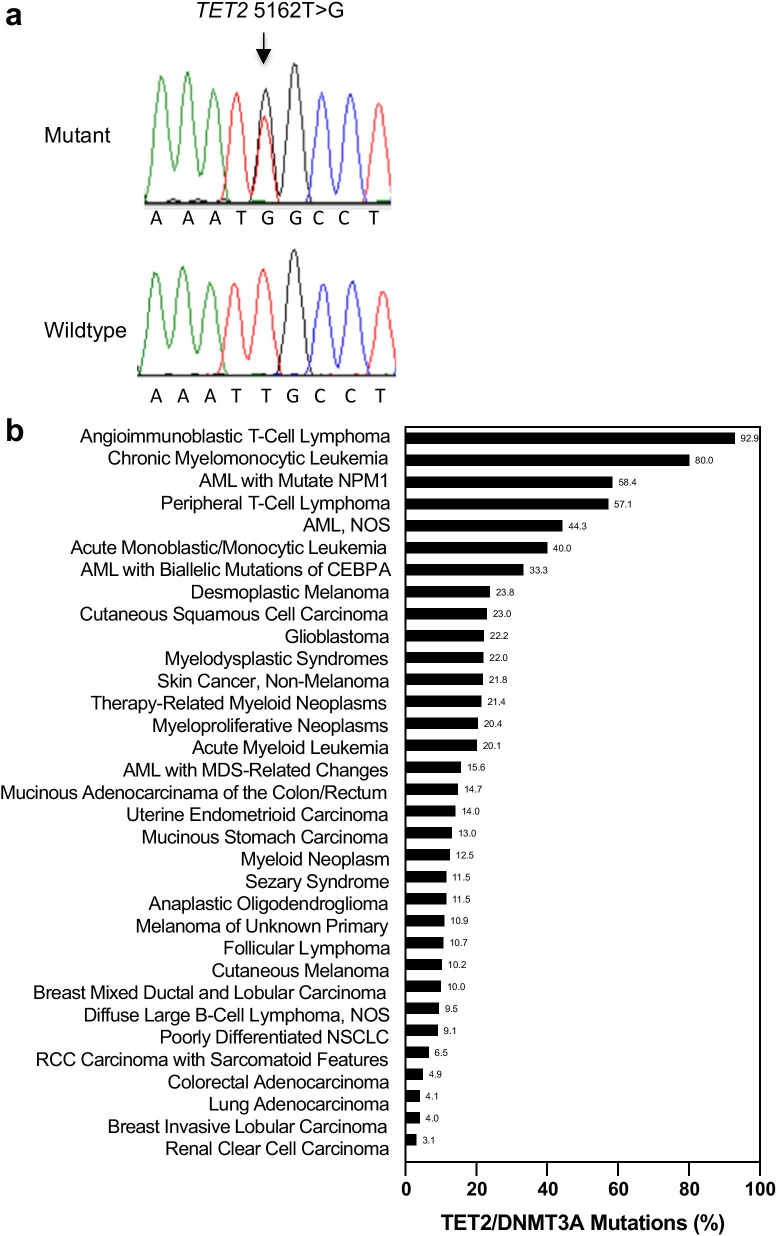
Validation of TET2 mutation in NCI-H23 cells by Sanger sequencing, and frequency of TET2 and/or DNMT3A mutations in human malignancies: **a** TET2 c.5162T > G missense mutation (upper panel; arrow) in NCI-H23 cells versus no alteration at the site in HCT116 cells (lower panel). Shown were all forward strands. **b** Frequency of TET2 and/or DNMT3A mutations in human malignancies analyzed from cBioPortal. AML, acute myeloid leukemia; CEBPA, CCAAT/enhancer-binding protein alpha; NPM, nucleophosmin; NSCLC, non-small cell lung cancer; NOS, not otherwise specified

The cell lines studied in vitro and in vivo were subjected to WES analysis as described in Methods. We revealed a novel deleterious *TET2* c.5162T > G p.L1721W missense mutation, along with confirmation of the *DNMT3A* c.667G > T (p.G223X) nonsense mutation in NCI-H23 cells (Table 1). Notably, the deleterious *TET2* L1721W missense mutation was recurrent and found in 35.3% (6/17) of cancer lines examined. In addition, deleterious *TET2* p.S477F (c.1430C > T) and nonsynonymous *DNMT3A* missense mutations were identified in SKMEL5 cells. Such mutation pattern was also observed in BT549 and ACHN cells but not in other 13 lines (Table 1). The *TET2* c.5162T > G mutation in NCI-H23 cells was validated by Sanger sequencing (Fig. 1a).

**Table 1:** *TET2* and *DNMT3A* mutation status in cancer cell lines

Cell line	*DNMT3A*		*TET2*	
	Mutation status; type^a^	Nucleotide; protein	Mutation status; type	Nucleotide; protein
ACHN	Missense; deleterious	c.2069T > C; p.V690A	Missense; deleterious	c.1088C > T; p.P363L
			Missense; deleterious	c.5162T > G; p.L1721W
BT549	Missense; deleterious	c.2587G > A; p.E863K	Missense; deleterious	c.1064G > A; p.G355D
			Missense; deleterious	c.4117G > A; p.A1373T
SKMEL5	Missense	c.736G > T; p.A246S	Missense; deleterious	c.1430C > T; p.S477F
NCI-H23	Nonsense	c.667G > T; p.G223X	Missense; deleterious	c.5162T > G; p.L1721W
SKMEL2	Wildtype^b^	–	Missense; deleterious	c.5162T > G; p.L1721W
DU145	Wildtype	–	Missense; deleterious	c.5162T > G; p.L1721W
KM12	Missense; deleterious	c.2283G > A; p.M761I	Missense	c.5284A > G; p.I1762V
OVCAR3	Wildtype	–	Wildtype	–
COLO205	Wildtype	–	Wildtype	–
MDA231	Wildtype	–	Missense; deleterious	c.5162T > G; p.L1721W
MCF7	Wildtype	–	Missense; deleterious	c.86C > G; p. P29R
M14	Wildtype	–	Wildtype	–
EKVX	Missense; deleterious	c.2375G > A; p.R792H	Missense	c.5284A > G; p.I1762V
T47D	Wildtype	–	Missense; deleterious	c.5162T > G; p.L1721W
SKOV3	Wildtype	–	Wildtype	–
HCT116	Wildtype	–	Missense	c.5284A > G; p.I1762V
HCT15	Missense; deleterious	c.2283G > A; p.M761I	Missense	c.5284A > G; p.I1762V

^a^Predicted by SIFT (sorting intolerant from tolerant) and/or PolyPhen (polymorphism phenotyping)

^b^Wildtype also refers to the synonymous variants

To investigate whether *TET2/DNMT3A* mutations were detectable in human tumor samples, we analyzed frequency of *TET2* and *DNMT3A* mutations in a spectrum of over 100 cancer types. The mutation rates for *DNMT3A* and *TET2* in either gene or both were as high as 92.9% in angioimmunoblastic T cell lymphoma, 80% in chronic myelomonocytic leukemia, 58.4% AML with mutated NPM1 and 57.1% in peripheral T cell lymphoma (Fig. 1b). The genes were also frequently mutated in several other types of leukemias, desmoplastic melanoma and cutaneous squamous cell carcinoma, glioblastoma and myelodysplasia. The alterations were found in 10.2% of 1241 cutaneous melanoma, 10% of 80 cases of breast mixed ductal and lobular carcinoma, 4.9% of 933 colorectal adenocarcinoma, and 4.1% of 2268 lung adenocarcinomas as well as 3.1% of 722 cases of renal clear cell carcinoma (Fig. 1b). The data suggest that *TET2/DNMT3A* mutations were present in many types of human malignancies and frequent in T cell lymphomas, chronic myelomonocytic leukemia and AML.

### *TET2*/*DNMT3A* mutations and T-dCyd antitumor activity in cells and animal models

**Fig. 2:**
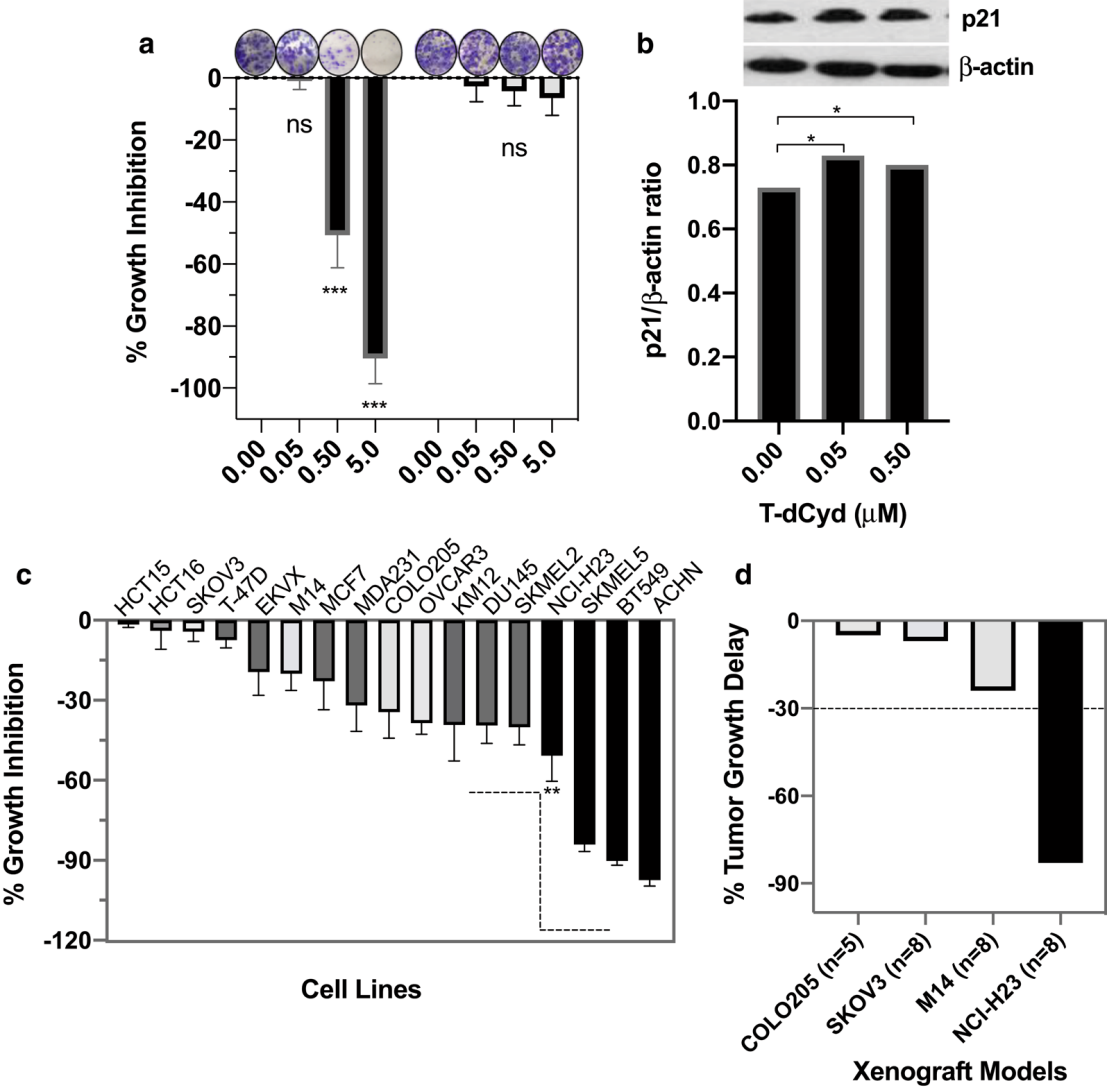
Cell and tumor growth inhibition by T-dCyd treatment in multiple cancer cell lines and xenograft tumor models with and without deleterious TET2 and nonsynonymous DNMT3A mutations: **a** The cell growth inhibition by increasing concentrations, as indicated, of T-dCyd in NCI-H23 cells with TET2/DNMT3A mutations (black bars) and wildtype SKOV3 cells (gray bars), ***P < 0.0001 compared to vehicle treatment; ns, not significant (left panel). **b** p21 expression in NCI-H23 cells treated with T-dCyd at 48 h by Western blotting (right panel), *P < 0.05 compared to vehicle. **c** Cell growth inhibition by 0.5 μM of T-dCyd evaluated by clonogenic assay in mutant groups (black), wildtype group (gray) and other status (dark gray), **P = 0.007 compared between black bar group and gray/dark-gray bar groups. **d** Tumor growth delay [(T-C)/C] in the mutant xenograft tumors (black), and wildtype tumors (gray) by T-dCyd treatment (all dosed with 4 mg/kg of T-dCyd). Dotted line indicated 30% of tumor growth delay. C, control; ns, not significant; T, treatment

To test our hypothesis that gene mutations in the epigenetic regulatory network were associated with T-dCyd antitumor activity, a panel of 17 cancer lines was analyzed by clonogenic assay. There was a dose-dependent inhibition of growth in co-mutant NCI-H23 and little activity in wildtype SKOV3 cells (Fig. 2a). Similar dose-dependent inhibition was noted in other cell lines with double-mutations and, to a lesser degree, in those with other patterns of alteration (Additional file 1: Fig. S1). To explore the mechanisms of growth inhibition by T-dCyd, expression of p21 was examined in NCI-H23 cells by Western blotting and immunocytochemistry (data not shown). p21 was increased by T-dCyd treatment (Fig. 2b), which was accompanied by TET2 inhibition and G2/S cell cycle arrest in NCI-H23 but not SKOV3 cells (Additional file 1: Fig. S1). When treated with 0.5 µMT-dCyd, growth inhibition was minimal to ~ 40% in 13 cell lines with wildtype and other status (DNMT3A wildtype/TET2 mutations, and DNMT3A mutations/TET2 p.I1762V mutation), and ~ 51% to 97.5% inhibition in 4 cell lines with deleterious *TET2* and nonsynonymous *DNMT3A* mutations (*P* = 0.007; Fig. 2c). Noticeably, *DNMT3A* mutations in coupling with non-deleterious *TET2*p.I1762V mutation were associated with lower activity of antitumor cells (Table 1 and Fig. 2c).

Subcutaneous xenograft models were established in nude mice to validate the in vitro findings. Animals were randomized to T-dCyd at 4 mg/kg or saline treatment as described in Methods. Mice with wildtype tumors including M14, SKOV3 and COLO205 had growth delay of < 25% after T-dCyd administrations (Fig. 2d). In contrast, average growth delay was 83% in mice bearing NCI-H23 tumors. T-dCyd treatment had no significant effect on the weight in the NCI-H23 xenograft mice (mean net weight loss 3.2% by T-dCyd vs. 0.2% in the control group) and no treatment-related death.

### Pharmacodynamic effects of T-dCyd in mutant xenograft tumors

**Fig. 3:**
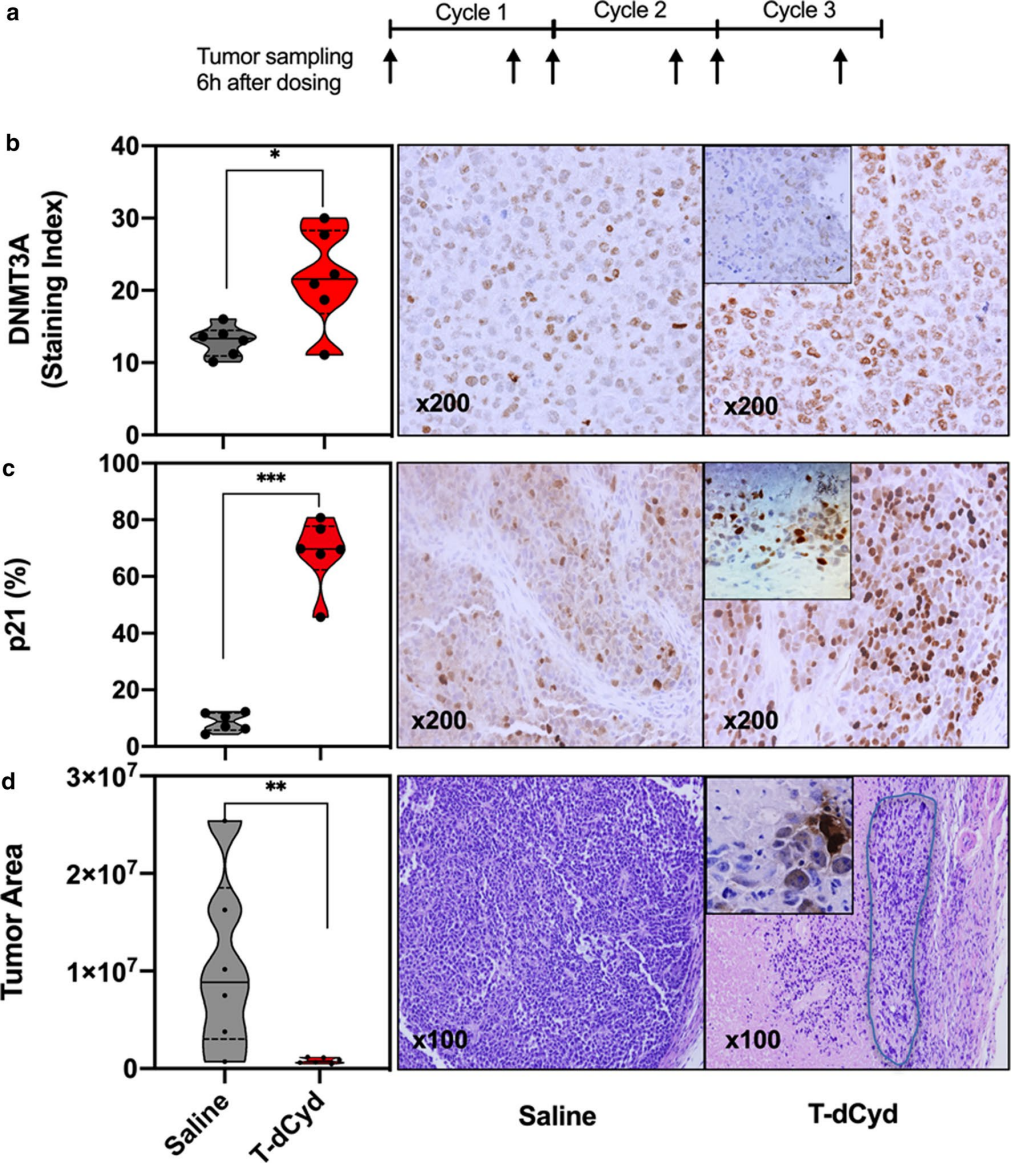
Effects of T-dCyd on NCI-H23 xenograft tumors in mice treated with 4 mg/kg T-dCyd and saline by immunohistochemical and microscopic analyses (6 mouse tumor samples per group): **a** The timeline of xenograft tumor sample collection as indicated by arrows. **b** Modulation of DNMT3A at C1D5 by T-dCyd versus saline shown in violin plot^a^ (left panel), *P < 0.05. Representative images of DNMT3A in the tumors treated by saline and T-dCyd at C1D5 (right panel), and the inset is a representative DNMT3A image at C3D5 (original magnification × 200). **c** Modulation of p21 at C1D5 by T-dCyd compared with saline shown in violin plot^a^ (left panel), ***P < 0.001. Representative images of p21 in the tumors treated by saline and T-dCyd at C1D5 (right panel), and the inset is a representative p21 image at C3D5 (× 200). **d** Measurement of the tumor areas on H&E sections quantified by digital imaging instrument in the T-dCyd group compared to saline group at C3D5 in the violin plot^a^ (left panel), **P < 0.01. Representative H&E sections of saline- and T-dCyd-treated tumor samples at C3D5 (right panel). Note a small solitary residue tumor lesion in the light blue circle with the presence of a bit of apoptosis (Inset; × 200) at end of T-dCyd treatment. ^a^Note: the solid band in the violin plot is median, upper or lower quartile (dotted line) represent 25% of data greater or less than this value, top and bottom borders of the violin plot are maximal and minimal values; and each dot represents an individual data point. C, cycle; D, day

To evaluate the molecular response to T-dCyd treatment, mice bearing NCI-H23 xenografts were subsequently established and treated with both 4 mg/kg and 2 mg/kg of T-dCyd or saline as a treatment schedule described in Methods. Tumor samples were collected at different time points as indicated in Fig. 3a. The harvested tumors were quartered, in which one piece was formalin-fixed and paraffin-embedded and the cut-sections were stained with H&E for tumor confirmation and used to assess the T-dCyd pharmacodynamics.

The effects of T-dCyd on p21, TET2 and DNMT3A were examined by immunohistochemistry in paraffin-embedded NCI-H23 xenograft tumors. TET2 expression was significantly inhibited by T-dCyd dosed at both 4 mg/kg and 2 mg/kg, respectively, at end of first cycle (C1D5), and the inhibition was sustainable at C3D5 (Table 2; Additional file 1: Table S1). It seemed that DNMT3A was increased at C1D5 but not sustainable at C3D5, likely due to a compensatory mechanism for a temporary increase (Table 2; Fig. 3b). Remarkably, nuclear p21 was increased by T-dCyd treatment (68.4% vs 8.7% by saline at C1D5, *P* = 0.0003; Table 2; Fig. 3c). The incremental increases were observed by subsequent dosing and reached 85.9% at the end of cycle 3 (*P* < 0.0001). Similarly, p21 was significantly augmented after drug administrations in the group dosed at 2 mg/kg of T-dCyd (P < 0.0001; Additional file 1: Table S1, Fig. S2a).

**Table 2:** Modulation of TET2, DNMT3A and p21 by T-dCyd (4 mg/kg) treatment in vivo

Target, mean ± SD	Saline^a^	T-dCyd			
		C1D5	*P* value^b^	C3D5^c^	*P* value^b^
TET2	42.7 ± 3.0	17.3 ± 6.7	< 0.0001	12.8 ± 6.0	< 0.0001
DNMT3A	13.0 ± 2.1	21.8 ± 6.7	0.02	10.6 ± 7.5	0.52
p21	8.7 ± 3.3	68.4 ± 12.2	0.0003	85.9 ± 6.1	< 0.0001

^a^Immunohistochemistry data from saline-treated samples at C1D1 were used and shown since p21, DNMT3A and TET2 immunohistochemistry results were similar at C1D1 and C3D5 by saline treatment

^b^Compared to saline group by unpaired *t* test

^c^There were five instead of 6 tumor samples available for analyses of TET2 and DNMT3A at C3D5. C,

cycle, D, day

Tumor areas were measured on H&E sections of the T-dCyd- and saline-treated samples collected at C3D5 by a digital imaging system (DAKO). Consistent with the measurement during therapy, digital quantitation confirmed that T-dCyd treatment resulted in a dramatic tumor reduction with an average of ~ 74% of smaller tumor areas than saline-treated controls (P < 0.01; Fig. 3d). Fewer residue tumor cells including a nugget of apoptosis and mostly non-tumor and necrotic components were present in the T-dCyd-treated tumor samples by microscopy. By contrast, saline-treated lesions were densely packed with tumor cells (Fig. 3d). In addition, significant reduction of the tumor areas with a few tumor cells was achieved in the group dosed by T-dCyd at 2 mg/kg level (Additional file 1: Fig. S2b).

## Discussion

In this study, we identified a novel deleterious *TET2* missense mutation at L1721W in NCI-H23 cells, not recorded in the COSMIC database, using WES technology and confirmed by Sanger sequencing. *TET2* L1721W mutation was recurrent in cancer cell lines derived from carcinomas of the breast, NSCLC, melanoma, renal, prostate, and was reportedly detected in patients with MDS [10]. *TET2* and/or *DNMT3A* was mutated in human solid tumors as well as in hematological malignancies to a greater degree (Fig. 1b). As such, they were most frequently mutated in T cell lymphomas, chronic myelomonocytic leukemia and AMLs. The alterations also occurred in desmoplastic melanoma, cutaneous squamous cell carcinoma and MDS. These mutations struck relatively frequent in myeloproliferative neoplasms and mucinous adenocarcinoma of the colon and rectum; they were detectable in cutaneous melanoma, breast mixed ductal and lobular carcinoma, renal clear cell carcinoma with sarcomatoid features and lung adenocarcinomas.

We demonstrated an association between the *TET2/DNMT3A* mutations and growth inhibition of cancer cells by T-dCyd treatment across 17 human solid tumor cell lines. Significant inhibition was achieved in cancer cells and animals bearing tumors that harbor deleterious *TET2* and nonsynonymous *DNMT3A* mutations. In contrast, those without such pattern of alterations were less responsive to treatment both in vitro and in vivo. It is worth noting that all *DNMT3A* mutations, except those co-occurring with non-deleterious TET2p.I1762V mutation, were associated with significant T-dCyd antitumor activities. Remarkably, T-dCyd given at 4 mg/kg nearly eradicated the tumor cells in NCI-H23 xenograft tumors at the end of treatment. *DNMT3A* mutation was shown to be associated with response to decitabine and azacytidine therapy in myeloid malignancies, and TET2 mutation was associated with the objective response to these DNMT inhibitors in MDS [6, 14].

We found an increase in p21, relative to DNA breakage/apoptosis (Additional file 1: Fig. S3), and G2/S cell cycle arrest by T-dCyd in *TET2/DNMT3A*-mutant NCI-H23 cells. Correspondingly, p21 was dramatically increased in NCI-H23 xenograft tumors by T-dCyd at effective doses. DNMT3A regulates p21 expression as a transcriptional co-repressor rather than through its DNA methyltransferase activity [2]. The mutant *DNMT3A* may have an impaired ability to bind to the transcriptional repression complex, thus abridging its transcriptional repression to p21 [4]. Reportedly, silencing DNMT3A led to an increase in p21 upon DNA damaging agent challenge [26–29]. Importantly, the incremental upregulation of p21 by T-dCyd dosing was accompanied by a remarkable arrest of tumor growth in the mutations-positive tumors in vivo. Additionally, T-dCyd inhibited TET2 in the double-mutant NCI-H23 cells and xenograft tumors (Additional file 1: Fig. S4). Thus, DNMT3A alteration in conjunction with deleterious TET2 mutation was critical to T-dCyd antitumor effect.

## Conclusions

We demonstrated that co-occurrence of *TET2* and *DNMT3A* mutations was present in human solid tumor cell lines and many types of human malignancies. Cell lines and animal model with deleterious *TET2* and nonsynonymous *DNMT3A* mutations were sensitive to T-dCyd treatment. The deleterious *TET2* c.5162T > G p.L1721W missense mutation is novel in NCI-H23 cells. The data confirmed our hypothesis that DNMT3A and TET2 gene alterations in the epigenetic regulatory network were critical to T-dCyd antitumor activity. Therefore, our preclinical data provide a promise to selectively treat cancer patients whose tumors carry deleterious *TET2* and nonsynonymous *DNMT3A* mutations.

## Supplementary Information

**Additional file 1: Fig. S1**. Inhibition of growth and TET2, and cell cycle arrest by T-dCyd in human solid tumor cell lines. **Fig. S2**. Effects of T-dCyd on DNMT3A/TET2-mutant NCI-H23 xenograft tumors in mice treated with 2 mg/kg T-dCyd by immunohistochemical and microscopic analyses (6 tumor samples per group). **Fig. S3**. Effects of T-dCyd on the induction of apoptosis in NCI-H23 cells. **Fig. S4**. Effects of T-dCyd on TET2 expression in DNMT3A/TET2-mutant NCI-H23 xenograft tumors.

## Data Availability

The data that support the findings of this study are available from the authors upon reasonable request and with permission of the National Cancer Institute.

## Abbreviations

AML: Acute myeloid leukemia
azacytidine: 5-Azacytidine
CDKN1A: Cyclin-dependent kinase inhibitor 1A
COSMIC: Catalogue of Somatic Mutations in Cancer
decitabine: 5-Aza-2′-deoxycytidine
DNMT: DNA methyltransferase
kg: Kilogram
MDS: Myelodysplastic syndromes
NSCLC: Non-small cell lung cancer
TET 2: Ten-Eleven Translocation methylcytosine dioxygenase 2
T-dCyd: 4′-Thio-2′-deoxycytidine
TUNEL: TdT-mediated dUTP nick-end labeling
WES: Whole exome sequencing

## References

[1] Okano M, Bell DW, Haber DA, Li E (1999). DNA methyltransferases Dnmt3a and Dnmt3b are essential for de novo methylation and mammalian development. Cell.

[2] Fuks F, Burgers WA, Godin N, Kasai M, Kouzarides T (2001). Dnmt3a binds deacetylases and is recruited by a sequence-specific repressor to silence transcription. EMBO J.

[3] Bachman KE, Rountree MR, Baylin SB (2001). Dnmt3a and Dnmt3b are transcriptional repressors that exhibit unique localization properties to heterochromatin. J Biol Chem.

[4] Ley TJ (2010). DNMT3A mutations in acute myeloid leukemia. N Engl J Med.

[5] Im AP (2014). DNMT3A and IDH mutations in acute myeloid leukemia and other myeloid malignancies: associations with prognosis and potential treatment strategies. Leukemia.

[6] Metzeler KH (2012). DNMT3A mutations and response to the hypomethylating agent decitabine in acute myeloid leukemia. Leukemia.

[7] DiNardo CD (2014). Lack of association of IDH1, IDH2 and DNMT3A mutations with outcome in older patients with acute myeloid leukemia treated with hypomethylating agents. Leuk Lymphoma.

[8] Pastor WA, Aravind L, Rao A (2013). TETonic shift: biological roles of TET proteins in DNA demethylation and transcription. Nat Rev Mol Cell Biol.

[9] Rasmussen KD (2015). Loss of TET2 in hematopoietic cells leads to DNA hypermethylation of active enhancers and induction of leukemogenesis. Genes Dev.

[10] Langemeijer SM (2009). Acquired mutations in TET2 are common in myelodysplastic syndromes. Nat Genet.

[11] Delhommeau F (2009). Mutation in TET2 in myeloid cancers. N Engl J Med.

[12] Quivoron C (2011). TET2 inactivation results in pleiotropic hematopoietic abnormalities in mouse and is a recurrent event during human lymphomagenesis. Cancer Cell.

[13] Koboldt DC (2016). Rare variation in TET2 is associated with clinically relevant prostate carcinoma in African Americans. Cancer Epidemiol Biomarkers Prev.

[14] Bejar R (2014). TET2 mutations predict response to hypomethylating agents in myelodysplastic syndrome patients. Blood.

[15] Couronne L, Bastard C, Bernard OA (2012). TET2 and DNMT3A mutations in human T-cell lymphoma. N Engl J Med.

[16] Bond J (2019). DNMT3A mutation is associated with increased age and adverse outcome in adult T-cell acute lymphoblastic leukemia. Haematologica.

[17] Zhang X (2016). DNMT3A and TET2 compete and cooperate to repress lineage-specific transcription factors in hematopoietic stem cells. Nat Genet.

[18] Thottassery JV (2014). Novel DNA methyltransferase-1 (DNMT1) depleting anticancer nucleosides, 4'-thio-2'-deoxycytidine and 5-aza-4'-thio-2'-deoxycytidine. Cancer Chemother Pharmacol.

[19] Cerami E (2012). The cBio cancer genomics portal: an open platform for exploring multidimensional cancer genomics data. Cancer Discov.

[20] Yang SX (2010). Akt phosphorylation at Ser473 predicts benefit of paclitaxel chemotherapy in node-positive breast cancer. J Clin Oncol.

[21] Nguyen D (2015). Notch1 phenotype and clinical stage progression in non-small cell lung cancer. J Hematol Oncol.

[22] Nguyen D, Yu J, Reinhold WC, Yang SX (2020). Association of independent prognostic factors and treatment modality with survival and recurrence outcomes in breast cancer. JAMA Netw Open.

[23] Yang SX, Steinberg SM, Nguyen D, Swain SM (2011). p53, HER2 and tumor cell apoptosis correlate with clinical outcome after neoadjuvant bevacizumab plus chemotherapy in breast cancer. Int J Oncol.

[24] Nguyen D (2011). Poly(ADP-ribose) polymerase inhibition enhances p53-dependent and -independent DNA damage responses induced by DNA damaging agent. Cell Cycle.

[25] Hollingshead MG (2014). Gene expression profiling of 49 human tumor xenografts from in vitro culture through multiple in vivo passages–strategies for data mining in support of therapeutic studies. BMC Genomics.

[26] Jiemjit A (2008). p21(WAF1/CIP1) induction by 5-azacytosine nucleosides requires DNA damage. Oncogene.

[27] Shin DY (2013). Decitabine, a DNA methyltransferases inhibitor, induces cell cycle arrest at G2/M phase through p53-independent pathway in human cancer cells. Biomed Pharmacother.

[28] Zhang Y (2010). Chromatin methylation activity of Dnmt3a and Dnmt3a/3L is guided by interaction of the ADD domain with the histone H3 tail. Nucleic Acids Res.

[29] Yang L, Rau R, Goodell MA (2015). DNMT3A in haematological malignancies. Nat Rev Cancer.

