# Supplementary material for: TET2 and DNMT3A mutations and exceptional response to 4′-thio-2′-deoxycytidine in human solid tumor models
Source: J Hematol Oncol. 2021 May 26;14:83. doi: 10.1186/s13045-021-01091-5 (PMC8157655; doi:10.1186/s13045-021-01091-5)
Supplement: Supplementary file 1 — Additional file 1: Fig. S1. Inhibition of growth and TET2, and cell cycle arrest by T-dCyd in human solid tumor cell lines. Fig. S2. Effects of T-dCyd on DNMT3A/TET2-mutant NCI-H23 xenograft tumors in mice treated with 2 mg/kg T-dCyd by immunohistochemical and microscopic analyses (6 tumor samples per group). Fig. S3. Effects of T-dCyd on the induction of apoptosis in NCI-H23 cells. Fig. S4. Effects of T-dCyd on TET2 expression in DNMT3A/TET2-mutant NCI-H23 xenograft tumors. [file 13045_2021_1091_MOESM1_ESM.pdf]

Research

TET2 and *DNMT3A* mutations associated with exceptional response to 4’-thio-2'-deoxycytidine in human solid tumor models

Sherry X. Yang[1*], Melinda Hollingshead[1], Larry Rubinstein[1], Dat Nguyen[1], Angelo B. A. Larenjeira[1], Robert J. Kinders[2], Michael Difilippantonio[1] and James H. Doroshow[1]

[1]Division of Cancer Treatment and Diagnosis, National Cancer Institute, National Institutes of Health, Bethesda, Maryland, USA; [2]Leidos Biomedical Research, Inc., Frederick, MD, USA

Running title: *DNMT3A/TET2* mutations and 4’-thio-2'-deoxycytidine efficacy

Key words: DNMT3A and TET2 mutations, NCI-H23 cells, p21, 4’-thio-2'-deoxycytidine (T-dCyd), whole exome sequencing, xenograft tumors

***Correspondence**: sherry.yang@nih.gov

Table S1 Modulation of TET2, DNMT3A and p21 by T-dCyd (2 mg/kg) treatment in vivo

Target, mean ± SD	Saline [a]	T-dCyd			
		C1D5 [b]	*P* value [c]	C3D5	*P* value [c]
TET2	42.7±3.0	26.6±3.8	<0.0001	1.4±1.4	<0.0001
DNMT3A	13.0±2.1	23.7±3.9	<0.0001	NA	NA
p21	8.7±3.3	63.5±10.0	<0.0001	92.3±9.0	<0.0001

[a] Immunohistochemistry data from untreated control samples at C1D1 were used and shown since p21, DNMT3A and TET2 immunohistochemistry results were similar from untreated control at C1D1 and saline treatment at C1D5 and C3D5

[b] There were five instead of 6 tumor samples available for analysis of TET2 at C1D5

[c] Compared to saline group by unpaired *t* test. NA, samples not available. C, cycle, D, day

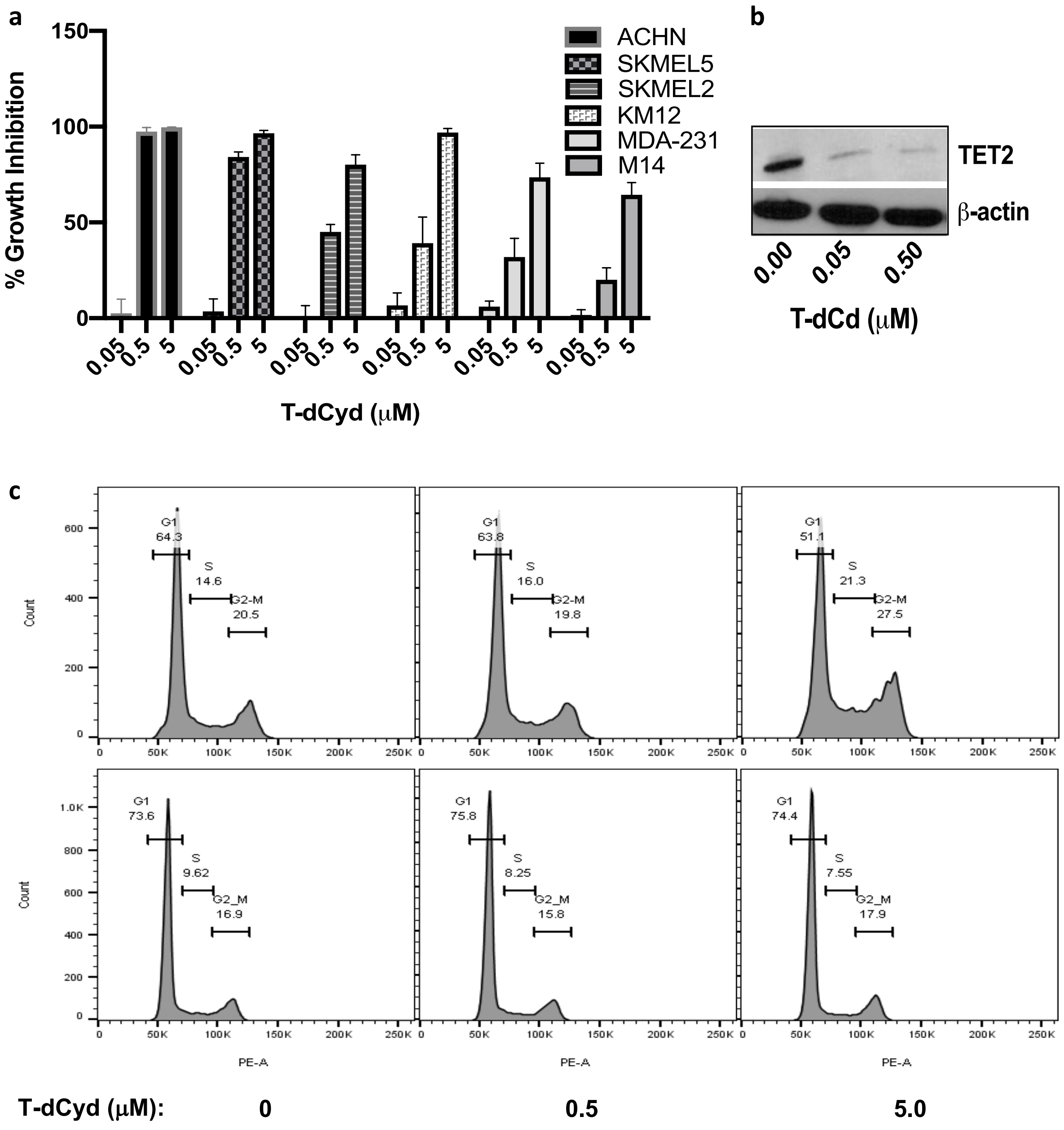

Fig. S1 Inhibition of growth and TET2, and cell cycle arrest by T-dCyd in human solid tumor cell lines. a Growth inhibition by T-dCd in representative cell lines. The dose-dependent growth inhibition, as indicated above, by T-dCyd in cell lines with various patterns of alteration of DNMT3A and TET2 (see Table 1) by clonogenic assay. **b** T-dCyd inhibited expression of TET2 in a dose-dependent manner in NCI-H23 cells. **c** G2/S cell cycle arrest by T-dCyd treatment for 24 h in NCI-H23 cells (upper panel) versus SKOV3 cells (lower panel). PE-A, propidium iodide excitation/emission-area

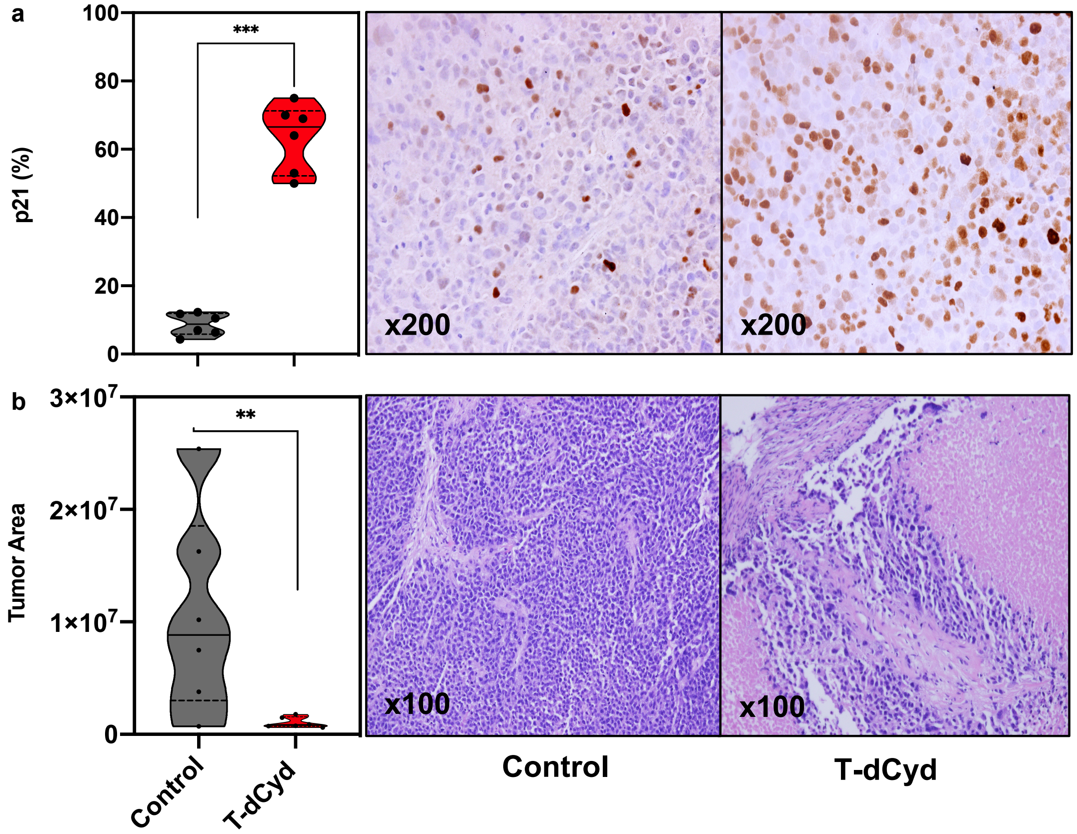

Fig. S2 Effects of T-dCyd on DNMT3A/TET2-mutant NCI-H23 xenograft tumors in mice treated with 2 mg/kg T-dCyd by immunohistochemical and microscopic analyses (6 tumor samples per group). a Modulation of p21 at C1D5 by T-dCyd versus control shown in violin plot[a] (left panel), ****P* < 0.001. Representative images of p21 of untreated control and T-dCyd treatment at the end of cycle 1 (right panel). **b** Measurement of the tumor area on H&E slides quantified using a digital imaging system in T-dCyd group compared to control group shown in the violin plot[a] (left panel), ***P* < 0.01. Representative images of H&E sections of control and T-dCyd-treated tumor samples at the end of treatment (C3D5; right panel). [a]Note: the solid band in the violin plot is median, upper or lower quartile (dotted line) represent 25% of data greater or less than this value, top and bottom borders of the violin are maximal and minimal values; and each dot represents an individual data point. C, cycle; D, day

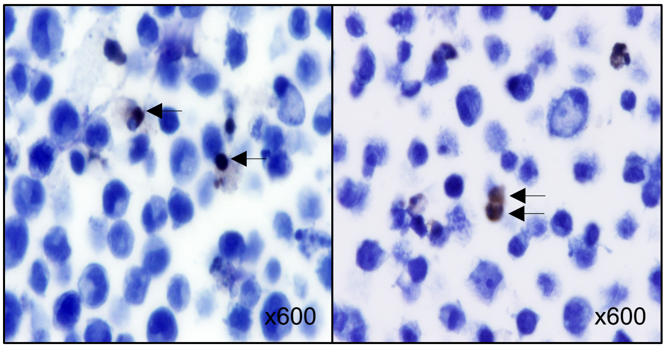

Vehicle **T-dCyd (1 μM)**

NCI-H23 Cells

Fig. S3 Effects of T-dCyd on the induction of apoptosis in NCI-H23 cells. The cells were treated with vehicle (left panel) and 1 μM of T-dCyd for 96 h (right panel), and DNA breakage/apoptosis was detected by TUNEL assay as described in the Method. Arrows indicate the cells under going apoptosis

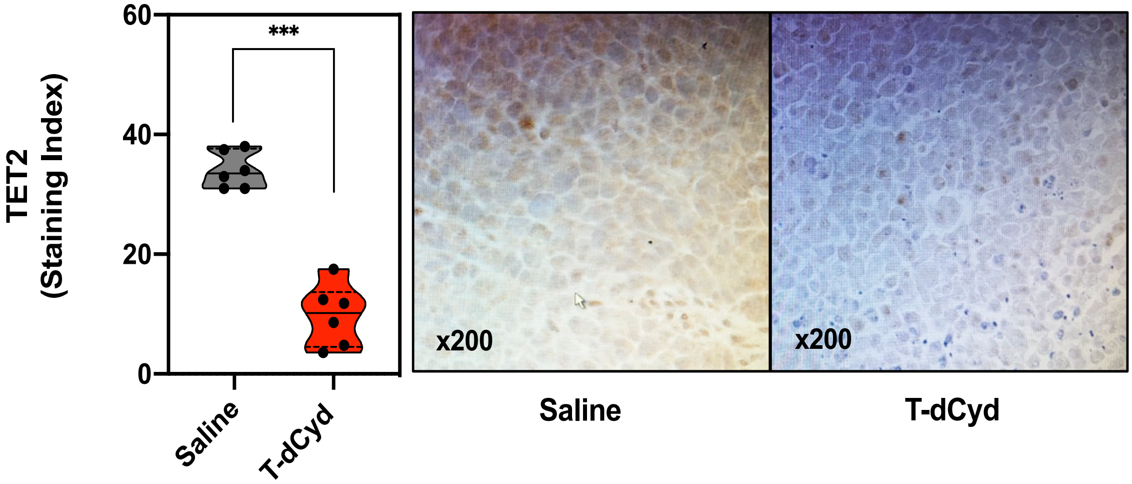

Fig. S4 Effects of T-dCyd on TET2 expression in DNMT3A/TET2-mutant NCI-H23 xenograft tumors. Mice were treated with 2 mg/kg T-dCyd for 14 days by daily dosing for a total of 2 cycles and TET2 expression was detected by immunohistochemical analyses. Modulation of TET2 at C1D14 by T-dCyd treatment compared to saline (C3D5) shown in violin plot[a] (left panel), ****P* < 0.001 by unpaired *t* test. Representative images of TET2 by saline and T-dCyd treatments at C1D14 (right panel). [a]Note: the solid band in the violin plot is median, upper or lower quartile (dotted line) represent 25% of data greater or less than this value, top and bottom borders of the violin plot are maximal and minimal values; and each dot represents an individual mouse data point. C, cycle; D, day
